# Catatonia and Systemic Lupus Erythematosus – A Case Report

**DOI:** 10.1192/bjo.2025.10717

**Published:** 2025-06-20

**Authors:** Natalie Da Silva, David Hall, Tobias Rowland

**Affiliations:** Coventry & Warwickshire Partnership NHS Trust, Coventry, United Kingdom

## Abstract

**Aims:** Catatonia is a complex neuropsychiatric syndrome of disturbed psychomotor function, abnormal behaviours and withdrawal. It remains under-recognised and under-diagnosed, especially within the acute hospital setting.

While often associated with primary mental illness, it can also occur secondary to systemic medical conditions such as systemic lupus erythematosus (SLE), an autoimmune disease in which neuropsychiatric manifestations are commonly described.

We present a case which highlights the diagnostic challenge and importance of recognising catatonia in the context of lupus.

**Methods:** A 34-year-old female with a three-year history of SLE presented with decline in functioning accompanied by malar rash, joint pains, paucity of speech and altered mental state. She had previously experienced command hallucinations in the context of lupus flares and though the psychotic component resolved between episodes, she was prescribed a daily maintenance dose of olanzapine 2.5 mg.

Assessment revealed an agitated, distracted patient with suspected auditory and visual hallucinations, profound paucity of speech and incoherent mumbling. She required assistance with personal care, displayed rigid posturing, and had stopped eating and drinking. Laboratory results were consistent with an acute SLE flare, and it was proposed that her presentation was SLE-related psychosis, initially addressed by increasing olanzapine dose with minimal effect.

Further clinical deterioration prompted a lumbar puncture after which the patient began to talk and regain some normal functioning. Thorough examination of notes revealed midazolam had been administered so it was proposed that this was catatonia and therefore partially resolved with a benzodiazepine. Further examination revealed waxy flexibility, catalepsy, stupor and mutism. Regular lorazepam was added to the schedule of cyclophosphamide and high-dose prednisolone and led to prompt substantial clinical improvement.

**Results:** Data suggests neuropsychiatric symptoms are common in SLE and though there are some reports in literature of lupus-associated catatonia, its precise prevalence is uncertain.

It is proposed that the diversity of symptoms can arise due to various pathophysiological mechanisms in lupus, which include autoimmune inflammation of the central nervous system, metabolic disturbances or adverse effects of medication. While treatment of the underlying cause is key, timely recognition of catatonia and pharmacological therapy can result in rapid clinical improvement.

**Conclusion:** Catatonia is associated with significant morbidity and mortality if untreated. It should be considered as a differential in patients with lupus, particularly those with concurrent neuropsychiatric symptoms, thus resulting in improved patient outcomes.

